# Associations of DNMT3B −149C>T and −2437T>A polymorphisms and lung cancer risk in Chinese population

**DOI:** 10.1186/s12957-016-1052-9

**Published:** 2016-11-22

**Authors:** Min Gao, Daqiang He, Fanji Meng, Jianing Li, Yan Shen

**Affiliations:** 1 Department of Neurology, the Fourth Hospital of Harbin Medical University, 37 Yiyuan Street, Harbin, Heilongjiang 150001 China; 2 Department of Radiology, the Fourth Hospital of Harbin Medical University, Harbin, Heilongjiang China; 3 Department of Cardiology, the Fourth Hospital of Harbin Medical University, Harbin, Heilongjiang China; 4 Department of Geriatrics, the Fourth Hospital of Harbin Medical University, Harbin, Heilongjiang China

**Keywords:** Methylation, Lung cancer, *DNMT3B*, Polymorphism, −149C>T, −2437T>A

## Abstract

**Background:**

*DNMT3B* polymorphisms are associated with the susceptibility of lung cancer. *DNMT3B* −2437T>A is a novel polymorphism, and its influence on the risk of lung cancer in Chinese was investigated in this study. In addition, effect of *DNMT3B* −149C>T polymorphism on lung cancer was also explored.

**Methods:**

Genotyping in subjects were performed by PCR-RFLP. Haplotype frequencies were estimated by estimating haplotype software. Adjusted odds ratios (ORs) with 95% confidence intervals (CIs) were calculated by unconditional logistic regression analysis.

**Results:**

Neither of the two polymorphisms was correlated with lung cancer (−149C>T: CT+TT vs CC: OR = 0.78, 95%CI, 0.57 to 1.05, *P* = 0.361; −2437T>A: AT+AA vs TT: OR = 0.99, 95%CI, 0.74 to 1.33, *P* = 0.168). In stratification analysis, T-allele carrier genotype of −149C>T polymorphism resulted in a reduced lung cancer risk at stage II, compared with CC (OR = 0.46, 95%CI, 0.28 to 0.77, *P* = 0.023). In haplotype analysis, when −149C/−2437T was used as reference, the other combined genotypes of the two polymorphisms had no significant effect on lung cancer risk (*P* > 0.05).

**Conclusions:**

The two *DNMT3B* polymorphisms are not correlated with lung cancer risk among Chinese population nor the haplotype of them.

## Background

Lung cancer is the major reason for death and is more frequent in men worldwide [1]. Although lung cancer incidence has a decreasing trend in several high-income countries due to a decreased smoking prevalence, the trend in global is increasing [2]. Reportedly, approximately 1,590,000 individuals have died from lung cancer in the world [3]. Moreover, the 5-year survival rate of lung cancer is low, which is about 15% of the newly diagnosed cases [4].

Accumulating evidence has demonstrated that genetic variations are associated with cancer risks, and several of them are proposed as biomarkers for cancer diagnosis [5, 6]. One hallmark of lung cancer is the alteration in DNA methylation patterns, and methylated CpG islands are suggested as biomarkers for diagnosis and detection of early cancer [7, 8]. DNA methylation mediated by DNA methyltransferases, such as DNMT3A and DNMT3B, has been identified as an important epigenetic mechanism for regulation of chromosomal stability [9]. DNMT3A and DNMT3B action as two de novo methyltransferases to target the un-methylated CpG sites on gene promoter, thus build a new methylation pattern [10]. These two DNMTs contribute to reprogramming of the epigenome in many cancer types such as lung cancer [11]. The *DNMT3B* gene is characterized by highly polymorphic, and 345 polymorphisms of this gene have been detected [9]. Reportedly, *DNMT3B* −149C>T polymorphism is highly related to the increased promoter activity [12]. A previous case-control study has demonstrated the hypothesis that T allele in *DNMT3B* −149C>T polymorphism is tightly related to the increased risk of lung cancer among non-Hispanic whites [13]. However, the sample size is relatively small (319 patients and 340 controls). Moreover, different populations could generate different results in other cancers [14, 15], thus we conducted this study among the Chinese population. Currently, other *DNMT3B* polymorphisms, such as −579G>T and −283T>C, are investigated to ascertain their influences on lung cancer [16, 17]. It is hypothesized that haplotypes of *DNMT3B* polymorphisms may regulate the susceptibility to lung cancer, and among the three *DNMT3B* polymorphisms (−149C>T, −579G>T, and −283T>C), the haplotype −283T/−579G is reported to have a decreased effect on the risk of lung adenocarcinoma [16].

Herein, we conducted this case-control study recruiting a total of 1286 Chinese participants (684 cases and 602 controls), to explore the effects of *DNMT3B* −149C>T polymorphism on the susceptibility of lung cancer in Chinese population. In addition, we identified a novel *DNMT3B* polymorphism, −2437T>A, and also explored its relationship with lung cancer risk. Furthermore, we investigated influence from haplotypes of the two *DNMT3B* polymorphisms on the risk of lung cancer, aiming to provide novel insights into mechanisms on lung cancer development regulated by *DNMT3B* gene.

## Methods

### Study population

A cohort of 1286 subjects (lung cases: 684; healthy control: 602) were enrolled at the Third Hospital of Harbin Medical University. Patients were hospitalized from September 2009 to March 2011 and confirmed to suffer from primary lung cancer by pathological diagnosis without any hereditary disease. Tumor stages were examined based on the tumor-node-metastasis (TNM) classification [18]. Staging examination was performed such as chest X-ray; bronchofiberscopy; and chest, abdomen, and head CT scan and bone scan. Those who had smoked five cigarettes 1 day for two or more than 2 years were considered as “smokers.”

### Genotyping procedures

A volume of 5-mL veinal blood was obtained from each patient and kept at 4 °C after natrium citricum anticoagulation. Genomic DNA was extracted from peripheral blood lymphocytes within 1 week by the proteinase K digestion-saturated sodium chloride salting out method [19]. Then the DNA purity was evaluated by NanoDrop spectrophotometer, through calculating 260/280 nm ratio [20]. PCR-RFLP (polymerase chain reaction-restriction fragment length polymorphism) method was used to perform the genotyping analysis of the two *DNMT3B* polymorphisms, according to the protocol of Lee et al. [16]. In brief, PCR reactions were carried out in a 20-μl reaction system, consisting of 100 ng genomic DNA, 2 μl 10× buffer (20 mmol/L MgCl_2_), 160 μmol/L dNTPs, 200 nmol/L of each primer, and 2 U of *Tag* polymerase (Promega). Primers for the *DNMT3B* −149C>T polymorphism were C74468A, 5′-GCCATATCAGTGAACCTTTAGAGAC-3′; G74582A, 5′-GGGG AGCACAATTTCCCTTC-3′; and for the −2437T>A polymorphism were C72555A, 5′-GGAACTGGAACTCAAGGCAAG-3′; T72687A, 5′- ACATGAATTATTGCTTATCG-3′. For −149C>T polymorphism, the third base in 3′ end of the forward primer was transferred from A to G, to create a Hinf I restrictive site; and for −2437T>A, the mutated base was “A” in the 3′ end of the forward primer, to generate a Tag I restrictive site. The PCR condition was initial denaturation at 94 °C for 5 min and then 35 cycles of the following procedures: 45 s at 94 °C, then 45 s at 58 °C for −149C>T and 45 s at 61.3 °C for −2437T>A, 45 s at 72 °C, a final elongation at 72 °C for 10 min.

The 286 bp PCR product of −149C>T was digested with 10 U Hinf I at 37 °C for 16 h, resolved on 4% acrylamide gel (8 μg/mL), and stained with ethidium bromide (EB) for visualization under UV light. Then polymorphism of *DNMT3B* −149C>T was genotyped basing on the band numbers: the CC genotype generates only one band (the entire 286 bp fragment), the TT produces two bands (245 and 23 bp), and heterozygote CT genotype yields three bands (286, 245, and 23 bp).

The 355 bp PCR product of −2437T>A was digested with 10 U Tag I at 37 °C for 16 h, resolved on 4% acrylamide gel, and stained with EB for visualization under UV light. The TT genotype produces one band (355 bp), AA genotype generates two bands (225 and 130 bp), and heterozygote TA genotype yields three bands (355, 225, and 130 bp).

For quality control, 10% of the individuals were randomly extracted to repeat the genotyping analysis. The genotyping results were 100% concordant. Additionally, three random PCR-amplified DNA samples for each genotype of the two polymorphisms were put through DNA sequencing, respectively, to determine the reliability of genotyping results, and the results were also 100% concordant.

### Statistical analyses

Differences in continuous variables are compared between control and lung cancer cases using the *χ* ^2^-test, such as allele frequency and genotype frequency distribution. Hardy-Weinberg equilibrium (HWE) was tested for genotype distributions of the control subjects [21]. Haplotype frequencies were estimated by the estimating haplotype software (http://linkage.rockefeller.edu/ott/eh.htm) [22]. The adjusted odds ratios (ORs) and 95% confidence intervals (CIs) by sex and age were determined with the unconditional logistic regression analysis. A *P* value <0.05 indicates the statistical significance. Subgroup analyses stratified by pathological type, TNM stage, and smoke status were performed. All the statistical analyses were performed by SPSS software (Chicago, IL, USA).

## Results

### Characteristics of subjects in the study

Main characteristics of the subjects are listed in Table 1. All patients have not received any anticancer therapy nor had the history of occupational exposure to carcinogenic factors. Tumor types were determined according to lung tumor tissue typing classification, and there were 221 (32.26%) squamous carcinoma cases, 257 (37.54%) adenocarcinoma cases, 142 (20.83%) small cell carcinoma, and 64 (9.37%) other carcinoma cases. A total of 239 (34.9%) patients were in stage I, 181 (26.5%) were in stage II, 235 (34.4%) were in stage III, and 29 (4.2%) were in stage IV. There were no obvious differences on the subject number distributions between the two kinds of samples with regard to gender and mean age. However, lung cancer cases had a significant higher frequency of smokers than controls (66.9 vs 44.6%), suggesting smoking was a causative factor for lung cancer risk.

**Table 1:** Baseline characteristics of subjects in lung cancer group and healthy control group case: 684; control: 602

	No. of cases (%)	No. of controls (%)	Allele frequency	
			*χ* ^2^	*P*
Gender				
Male	498 (72.8)	400 (66.5)	0.703	0.402
Female	186 (28.2)	202 (33.5)		
Age (*x* ± *s*)	56.1 ± 11.3	57.1 ± 10.0	1.306	0.192
<60	408 (59.7)	328 (54.5)	2.006	0.157
≥60	276 (40.3)	274 (45.5)		
Smoking status				
Smokers	458 (66.9)	268 (44.6)	37.461	0.001
Non-smokers	226 (33.1)	334 (55.4)		
Pathological type				
SCC	221 (32.3)	–		
AC	257 (37.6)	–		
ASC	64 (9.4)	–		
SCLC	142 (20.8)	–		
TNM staging				
I	239 (34.9)	–		
II	181 (26.5)	–		
III	235 (34.4)	–		
IV	29 (4.2)	–		
T2437A allele				
T	559 (69.2)	497 (70.6)	0.357	0.550
A	249 (30.8)	207 (29.4)		
C149T				
C	619 (76.6)	559 (79.4)	1.707	0.191
A	189 (23.4)	145 (20.6)		

*SCC* squamous cell carcinoma, *AC* adenomatous carcinoma, *ASC* adenosquamous carcinomas, *SCLC* small cell lung cancer, *TNM* tumor-node-metastasis

### Genotype frequency and associations between DNMT3B −149C>T and −2437T>A polymorphisms and risk of lung cancer

Genotype distributions of the two polymorphisms are shown in Table 2. For *DNMT3B* −149C>T polymorphism, when CC genotype was used as reference, the T-allele carrier genotypes (CT+TT) did not show any pronounced correlations with risk of lung cancer (OR = 0.78, 95% CI, 0.57 to 1.05, *P* = 0.361). Likewise, significant differences were detected in neither TT genotype (OR = 0.90, 95% CI, 0.62 to 2.37, *P* = 0.406) nor CT genotype (OR = 1.42, 95% CI, 0.34 to 1.53, *P* = 0.541), compared with CC. For *DNMT3B* −2437T>A polymorphism, when TT genotype was used as reference, the A-allele carrier genotypes (AT+AA) were not remarkably related to lung cancer risk (OR = 0.99, 95% CI, 0.74 to 1.33, *P* = 0.168). No significant differences were detected in AA genotype (OR = 1.22, 95% CI, 0.58 to 1.35, *P* = 0.215) or TA genotype (OR = 1.00, 95% CI, 0.42 to 1.67, *P* = 0.308), compared with TT genotype. In control subjects, the genotype frequency was in accordance with HWE expectation (*P* > 0.05).

**Table 2:** Genotype distribution of *DNMT3B* −149C>T and −2437T>A polymorphisms and their associations with lung cancer risk

Genotype	No. of cases (%)	No. of controls^a^ (%)	OR^b^ (95% CI)	*P* value
−149C>T				
TT	35 (5.1)	38 (6.3)	0.90 (0.62 2.37)	0.406
CT	249 (36.4)	172 (28.6)	1.42 (0.34 1.53)	0.541
CC	400 (58.5)	392 (65.1)	Reference	
T-allele carriers	284 (41.5)	210 (34.9)	0.78 (0.57 1.05)	0.361
−2437T>A				
AA	76 (11.1)	56 (9.3)	1.22 (0.58 1.35)	0.215
TA	269 (39.4)	241 (40.1)	1.00 (0.42 1.67)	0.308
TT	339 (49.5)	304 (50.6)	Reference	
A-allele carriers	345 (50.5)	297 (49.4)	0.99 (0.74, 1.33)	0.167

*No.* number of cases or controls, *OR* odds ratio, *CI* confidence interval

^a^The observed genotype distribution in controls was in accordance with the Hardy-Weinberg equilibrium (*P* = 0.153 for DNMT3B −149C>T and *P* = 0.187 for DNMT3B −2437T>A)

^b^Adjusted for age and sex in a logistic regression model

### Subgroup analysis

Subgroup analysis stratified by pathological type indicated that there were no significant associations between any of the two *DNMT3B* polymorphisms and the risk of any lung cancer type (Table 3).

**Table 3:** Subgroup analysis stratified by pathological type

Genotype	Pathological type							
	SCC	OR^a^ (95%CI)	AC	OR^a^ (95%CI)	ASC	OR^a^ (95%CI)	SCLC	OR^a^ (95%CI)
−149C>T								
TT	19 (8.5)	0.54 (0.31 1.56)	15 (5.8)	0.23 (0.33 2.01)	6 (9.8)	0.17 (0.54 1.98)	7 (4.8)	0.24 (0.31 2.12)
CT	63 (28.7)	0.76 (0.25 1.81)	81 (31.7)	0.81 (0.77 1.67)	22 (34.1)	0.42 (0.34 1.63)	37 (25.8)	0.58 (0.22 1.37)
CC	139 (62.8)	Reference	161 (62.5)	Reference	36 (56.1)	Reference	99 (69.4)	Reference
CT+TT	82 (37.2)	0.82 (0.53 1.27)	96 (37.5)	0.90 (0.59 1.39)	68 (.43.9)	0.55 (0.26 1.15)	44 (30.6)	0.63 (0.35 1.44)
−2437T>A								
AA	31 (14.0)	0.57 (1.21 2.14)	11 (4.2)	0.45 (0.72 1.51)	6 (9.8)	0.26 (1.32 2.31)	14 (9.7)	0.37 (0.87 1.92)
TA	79 (35.6)	0.85 (0.58 1.67)	122 (47.5)	0.89 (0.22 1.21)	22 (34.1)	0.76 (0.28 1.64)	55 (38.7)	0.88 (0.64 1.55)
TT	111 (50.4)	Reference	124 (48.3)	Reference	36 (56.1)	Reference	73 (51.6)	Reference
TA+AA	110 (49.6)	0.99 (0.65 1.52)	133 (51.7)	1.08 (0.71 1.64)	68 (43.9)	0.83 (0.43 1.61)	69 (48.4)	0.95 (0.56 1.64)

*SCC* squamous cell carcinoma, *AC* adenomatous carcinoma, *ASC* adenosquamous carcinomas, *SCLC* small cell lung cancer, *TNM* tumor-node-metastasis, *OR* odds ratio, *CI* confidence interval

^a^Adjusted for age and sex in a logistic regression model

With regard to TNM staging, only the CT+TT genotypes of −149C>T polymorphism showed a significant reduced lung cancer risk at the II stage, compared with the genotype of CC (OR = 0.46, 95% CI, 0.28 to 0.77, *P* = 0.023), while −2437T>A polymorphism was not related to risk of lung cancer risk at any stage (Table 4).

**Table 4:** Subgroup analysis stratified by different stages

Genotype	TNM staging							
	Stage I	OR^a^ (95% CI)	Stage II	OR^a^ (95%CI)	Stage III	OR^a^ (95% CI)	Stage IV	OR^a^ (95% CI)
−149C>T								
TT	23 (9.8)	0.57 (0.32 1.78)	4 (2.2)	0.23 (0.29 1.57)	12 (5.0)	0.32 (0.31 1.58)	4 (13.3)	0.75 (0.31 1.28)
CT	78 (32.5)	0.92 (0.54 1.52)	41 (22.5)	0.38 (0.42 1.81)	68 (28.9)	0.65 (0.38 1.85)	10 (33.3)	1.02 (0.82 2.54)
CC	138 (57.7)	Reference	136 (75.3)	Reference	155 (66.1)	Reference	15 (53.3)	Reference
CT+TT	101 (42.3)	1.03 (0.68 1.55)	45 (24.7)	0.46 (0.28 0.77)	80 (32.9)	0.72 (0.47 1.10)	14 (46.6)	1.23 (0.44 3.46)
−2437T>A								
AA	20 (8.1)	0.32 (0.28 1.35)	21 (11.8)	0.45 (0.28 2.51)	20 (8.3)	0.84 (0.81 1.95)	4 (13.3)	1.32 (0.82 1.69)
TA	91 (38.2)	0.72 (0.82 1.96)	66 (36.6)	0.78 (0.84 1.92)	101 (43.0)	0.97 (0.58 1.92)	15 (53.3)	1.56 (0.78 3.51)
TT	128 (53.7)	Reference	93 (51.6)	Reference	114 (48.7)	Reference	10 (33.3)	Reference
TA+AA	111 (46.3)	0.85 (0.57 1.27)	87 (48.4)	0.92 (0.59 1.44)	121 (51.3)	1.03 (0.69 1.55)	19 (66.6)	1.96 (0.66 5.84)

*OR* odds ratio, *CI* confidence interval

^a^Adjusted for age and sex in a logistic regression model

When stratified by smoke status, we found that compared with CC genotype of *DNMT3B* −149C>T polymorphism, none of the TT (smoker group: OR = 0.81, 95%CI, 0.84 to 2.35, *P* = 0.571; non-smoker group: OR = 1.59, 95% CI, 0.37 to 1.94, *P* = 0.371), CT (smoker group: OR = 0.74, 95% CI, 0.45 to 1.83, *P* = 0.469; non-smoker group: OR = 0.69, 95%CI, 0.24 to 1.64, *P* = 0.108), and TT+CT (smoker group: OR = 0.76, 95% CI, 0.52 to 1.17, *P* = 0.274; non-smoker group: OR = 0.77, 95% CI, 0.49 to 1.25, *P* = 0.251) indicated a significant difference in neither smoker group nor non-smoker group (Table 5). The OR values were newly the same in each comparison of the smoker group and the non-smoker group. For −2437T>A polymorphism, similar results were observed, and there were no significant differences between the allelic genotypes (AA, TA, and AA+TA) and TT genotype in smoker group or non-smoker group (*P* > 0.05, Table 5). Interestingly, we found that ORs of AA, TA, and AA+TA in smoker group were slightly higher than non-smokers (AA: 1.53 vs 0.71; TA: 1.78 vs 1.07; and AA + TA: 1.72 vs 1.00), which suggested that these genotypes might be more frequent in smokers than non-smokers.

**Table 5:** Subgroup analysis stratified by smoke status

Genotype	No. of cases (%)	No. of controls (%)	OR^a^ (95% CI)	*P* value
−149C>T				
Smokers				
TT	23 (5.1)	15 (5.6)	0.81 (0.84 2.35)	0.571
CT	135 (29.4)	95 (35.5)	0.74 (0.45 1.83)	0.469
CC	300 (65.5)	158 (58.9)	Reference	
CT+TT	158 (34.5)	110 (41.1)	0.76 (0.52 1.17)	0.274
Non-smokers				
TT	19 (8.5)	16 (4.9)	1.59 (0.37 1.94)	0.371
CT	62 (27.4)	124 (37.1)	0.69 (0.24 1.64)	0.108
CC	145 (64.1)	194 (58.0)	Reference	
CT+TT	81 (35.9)	140 (42)	0.77 (0.49 1.25)	0.251
−2437T>A				
Smokers				
AA	47 (51.9)	31 (51.6)	1.53 (0.42 1.97)	0.473
TA	173 (37.8)	98 (36.7)	1.78 (0.35 1.67)	0.082
TT	138 (10.2)	139 (11.7)	Reference	
TA+AA	220 (89.7)	129 (88.3)	1.72 (0.68 1.49)	0.627
Non-smokers				
AA	35 (47.9)	29 (47.8)	0.71 (0.34 1.68)	0.376
TA	204 (44.4)	111 (41.5)	1.07 (0.32 1.85)	0.723
TT	219 (7.7)	128 (10.7)	Reference	
TA+AA	239 (92.3)	140 (89.3)	1.00 (0.64 1.59)	0.504

*OR* odds ratio, *CI* confidence interval

^a^Adjusted for age and sex in a logistic regression model

### Haplotype analysis of the two polymorphisms

Four combined genotypes (149C/2437T, 149C/2437A, 149T/2437T, and 149T/2437A) were investigated between the two polymorphisms. When the genotypes of −149C/−2437T were used as the reference group, no remarkable correlation was detected between the other combined genotypes and risk of lung cancer (*P* > 0.05, Table 6).

**Table 6:** Association of DNMT3B −C149T and −T2437A haplotype with the risk of lung cancer

Haplotype	No. of controls (%)	No. of cases (%)	OR^a^ (95%CI)	*P* value
149C/2437T	428 (53.0)	295 (56.0)	Reference	
149C/2437A	191 (23.6)	164 (23.4)	0.93 (0.73, 1.19)	0.078
149T/2437T	131 (16.2)	102 (14.5)	0.84 (0.63, 1.13)	0.134
149T/2437A	58 (7.2)	43 (6.1)	0.80 (0.53, 1.22)	0.573

*No.* number of cases or controls, *OR* odds ratio, *CI* confidence interval

^a^Adjusted for age and sex in a logistic regression model

## Discussion

The present study investigated the association between two *DNMT3B* polymorphisms, −149C>T and −2437T>A, and lung cancer risk. As a result, neither of the two polymorphisms was at a significantly increased or decreased risk of lung cancer, when the wild genotypes (CC for 149C>T and TT for −2437T>A) were used as the reference groups. In the stratification analysis, T-allele carrier genotype of −149C>T polymorphism was closely related to decreased lung cancer risk at stage II, compared with the wild genotype (CC).

DNA methylation promotes the progression of cancer cells, and *DNMT3B* plays significant roles in hypermethylation and hypomethylation of genomic DNA [23]. Increased *DNMT3B* expression has been discovered in lung cancer [24]. *DNMT3B* −149C>T polymorphism is known to enhance the gene’s promoter activity, and a previous study has demonstrated that T-allele carrier genotypes, especially CT genotype, are significantly related to increased risk of lung cancer [13]. The possible explanation is the increased promoter activity of *DNMT3B* by this C to T transition might up-regulate genes that involve in regulation of methylation of some tumor suppressor genes [13]. Unfortunately, our results were inconsistent with this finding, and we did not detect any association. The different results may be due to different ethnic populations. Our study was conducted among Chinese population, while Shen’s is among the non-Hispanic whites. However, we enrolled more individuals in the present study and performed subgroup analysis stratified by lung cancer types and different tumor stages as well as smoke status. Notably, It is the first discovery that in stage II, T-allele carrier genotypes (CT and TT) of −149C>T polymorphism were highly related to the reduced risk of lung cancer. The plausible reason might be that gene expressions are different at different stages. For instance, *CDH13*, *RASSF1A*, and *APC* are identified as DNA methylation markers at stage I in non-small cell lung cancer (NSCLC), and methylation of these genes in the prompter region are associated with early recurrence [25], while methylation of *TFPI2* is mainly discovered in advanced stage (stage III) of NSCLC [26]. In addition, although methylation of several genes, such as *RARβ*, *TIMP-3*, *p16* ^*INK4a*^, *DAPK*, *p14* ^*ARF*^, and *GSTP1*, are frequently occurred in NSCLC, correlations between methylation changes of these genes in NSCLC tumors and the clinical data are different at different stages (stages I to III) [27]. Our findings indicated that *DNMT3B* −149C>T polymorphism might not be used as the prognostic marker for lung cancer, especially in Chinese population.

Several studies have reported the regulation or coordination of *DNMT3B* by other genes. For instance, it is common in cancer that the cell cycle-related genes, such as *p14* ^*ARF*^ and *p16* ^*INK4a*^, are inactivated. One major form of the epigenetic inactivation of *p14* ^*ARF*^ and *p16* ^*INK4a*^ is hypermethylation of CpG islands, and expressions of these two genes are tightly associated with increased expression of *DNMT3B* [28]. The gene *UHRF1*, which encodes a subfamily of RING-finger type E3 ubiquitin ligases, has an important role in DNA methylation maintenance [29]. In NSCLC, it is demonstrated that *UHRF1* controls cell cycle through silencing of tumor suppressor genes, and *DNMT3B* is also up-regulated in *UHRF1* knockdown clones [30]. These collectively suggest that *DNMT3B*’s role in lung cancer development might require involvement of other genes’ regulations. This provides a hint that in stage II, several potential suppressor genes might express and exert an inhibitory role on *DNMT3B* expression, despite the elevated prompter activity. However, this hypothesis needs to be further validated.

−2437T>A is a novel *DNMT3B* polymorphism that has never been reported before. In the present study, like −149C>T polymorphism, we did not find any association between A-allele carrier genotypes (AT+TT) and lung cancer risk, when CC was used as the reference group. Stratification analysis also indicated that there was no significant association. These findings suggest that although this polymorphism is novel, it might not influence the expression of *DNMT3B*, or there are more complicated mechanisms on the regulations of *DNMT3B* when this T to A transition was emerged.

To investigate associations between the combined influence of these two polymorphisms and lung cancer risk, the haplotype analysis was conducted and the result indicated that when −149C/−2437T was used as the reference, the other combined genotypes of the two polymorphisms were not significantly related to increased or decreased risk of lung cancer (*P* > 0.05). Nevertheless, other studies investigating haplotype in other *DNMT3B* polymorphisms reveal that −579G>T and −283T>C is a haplotype that could affect the *DNMT3B*’s expression, and the combined genotype −283T/−579G achieved a reduced risk of adenocarcinoma, in comparison with −283C/−579T [16]. However, results might be different in different lung cancer types. The present study indicated that the haplotype −149C>T and −2437T>A was not pronouncedly correlated with risk of lung cancer, suggesting the two polymorphisms could not be considered as a haplotype, during the progression of lung cancer.

The main limitation in the present study was that the control samples were hospital-based, which might cause selection bias. However, relevant data were adjusted by sex and age to reduce influences from such confounding factors and provide a reliable result. Additionally, interactions of *DNMT3B* and other genes were not investigated, which might affect the results. Nevertheless, our findings are of great value to provide a novel insight into effects of *DNMT3B* polymorphisms on risk of lung cancer among Chinese population.

## Conclusions

In conclusion, two *DNMT3B* polymorphisms, −149C>T and −2437T>A, are not related to lung cancer risk among Chinese population nor the haplotype of them. However, more studies with larger samples are required to confirm our findings.

## Abbreviations

CIs: Confidence intervals
EB: Ethidium bromide
HWE: Hardy-Weinberg equilibrium
NSCLC: Non-small cell lung cancer
ORs: Odds ratios
PCR-RFLP: Polymerase chain reaction-restriction fragment length polymorphism
TNM: Tumor-node-metastasis
